# Supplementary material for: Multi-omics analysis of green lineage osmotic stress pathways unveils crucial roles of different cellular compartments
Source: Nat Commun. 2024 Jul 16;15:5988. doi: 10.1038/s41467-024-49844-3 (PMC11252407; doi:10.1038/s41467-024-49844-3)
Supplement: Supplementary file 1 — Supplementary Information [file 41467_2024_49844_MOESM1_ESM.pdf]

Supplementary Figures

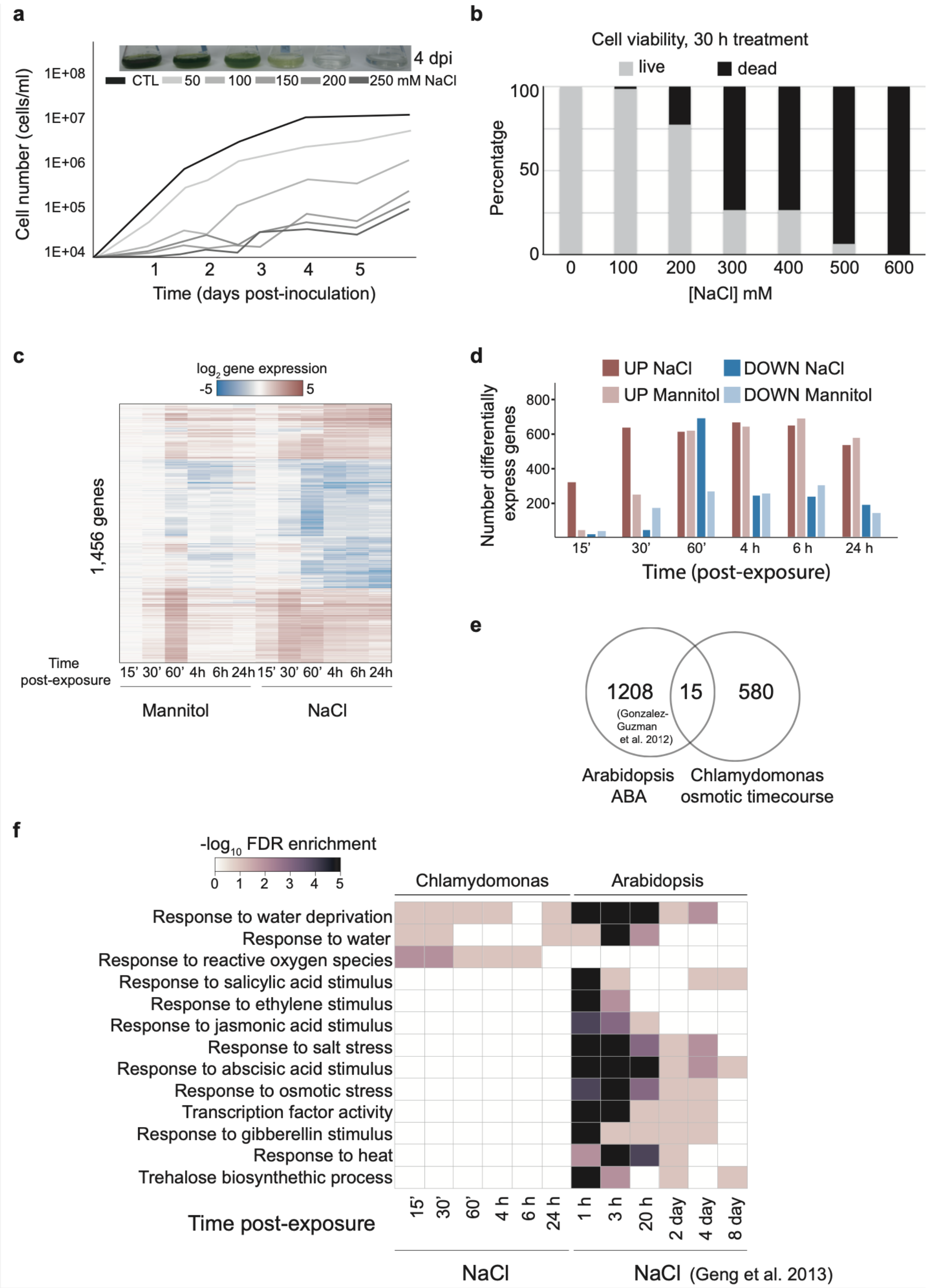

Supplementary Figure 1. *Chlamydomonas reinhardtii* transcriptional response to osmotic stress lacks obvious signs of ABA hormonal regulation. (Related to Figure 1, see also Supplementary Table 1-3).

A. Growth curves of Chlamydomonas wild-type cells (CC-4533) grown under different concentrations of NaCl.
B. Cell viability of Chlamydomonas wild-type cells treated with different concentrations of NaCl for 30 hours. Evans blue staining was used to assess cell viability.

C. Heat map representing clustered log2 values of 1,456 differentially regulated genes (FC > 2, FDR < 0.01) under 100 mM NaCl and 300 mM Mannitol treatment at different timepoints. (' and h indicates minutes and hours respectively). See Supplementary Table S2 for raw data.
D. Number of differentially expressed genes (FC > 2, FDR < 0.01) at different timepoints. (' and h indicates minutes and hours respectively).
E. Overlap of Chlamydomonas differentially expressed genes during NaCl and mannitol time course with Arabidopsis orthologous and differentially regulated genes upon ABA treatment in Arabidopsis. See Supplementary Table 2.
F. Gene ontology analysis of differentially regulated genes across the Chlamydomonas time course treated with 100 mM NaCl and NaCl time course of Arabidopsis roots. See Supplementary Table 2.

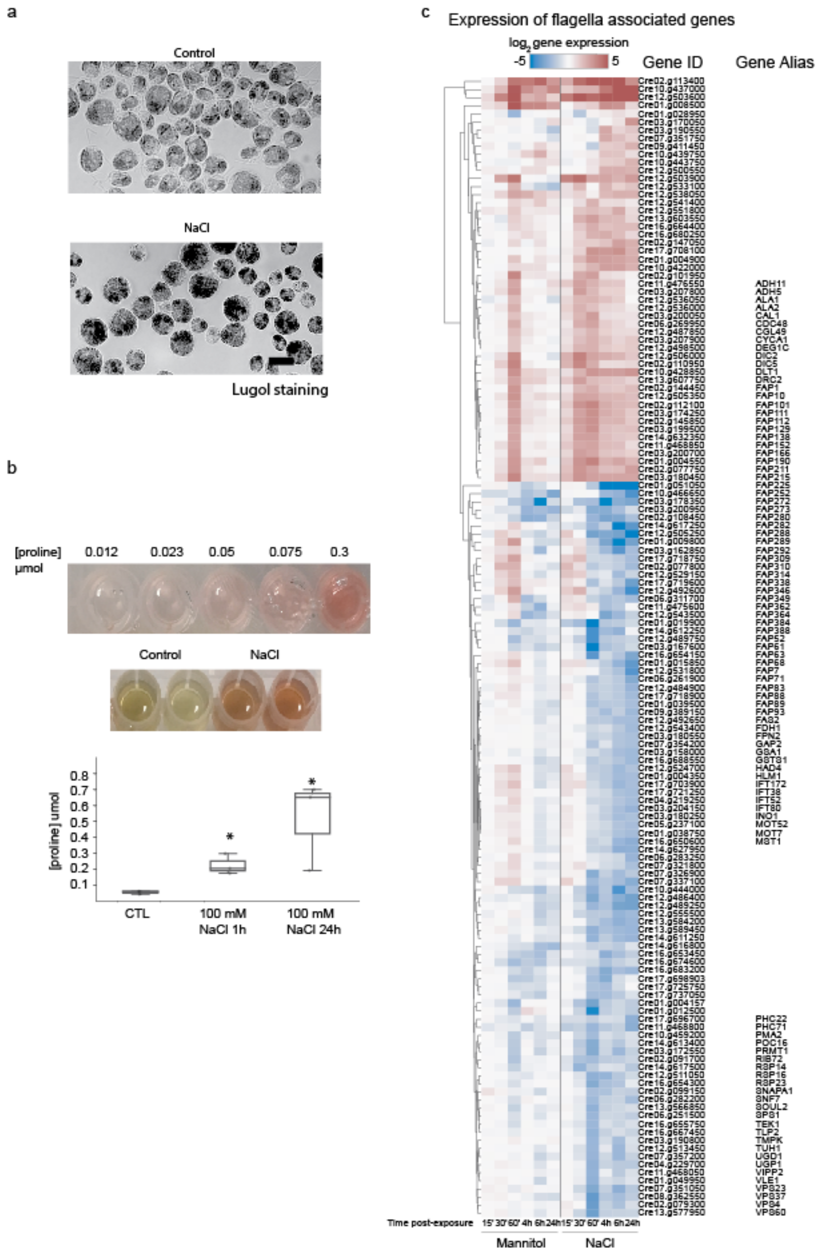

Supplementary Figure 2. Chlamydomonas osmotic responses to osmotic stress. (Related to Figure 1).

A. Chlamydomonas cells stained with Lugol. (Left) CTL; Control Chlamydomonas cells in mid-early exponential phase stained with Lugol. (Right) NaCl, Chlamydomonas cells in mid-early exponential phase treated with 100 mM NaCl for 20 hours and stained with Lugol. Scale bars = 10 μm.

B. Proline quantification of Chlamydomonas cells upon 100mM NaCl treatment for 16 hours. n=3 independent measurements from pooled cells, taken from three independent experiments. Center lines show medians and box limits indicate the 25th and 75th percentiles. Whiskers represent minina and maxima, * indicates significance, one-way ANOVA test between different conditions and control, p-value<0.05.

C. Heat map representing log2 gene expression of Chlamydomonas Ciliary proteins (The Chlamydomonas Flagellar proteome project (http://chlamyfp.org/index.php)) differentially regulated in the Chlamydomonas osmotic transcriptome. See also Supplementary Table 2.

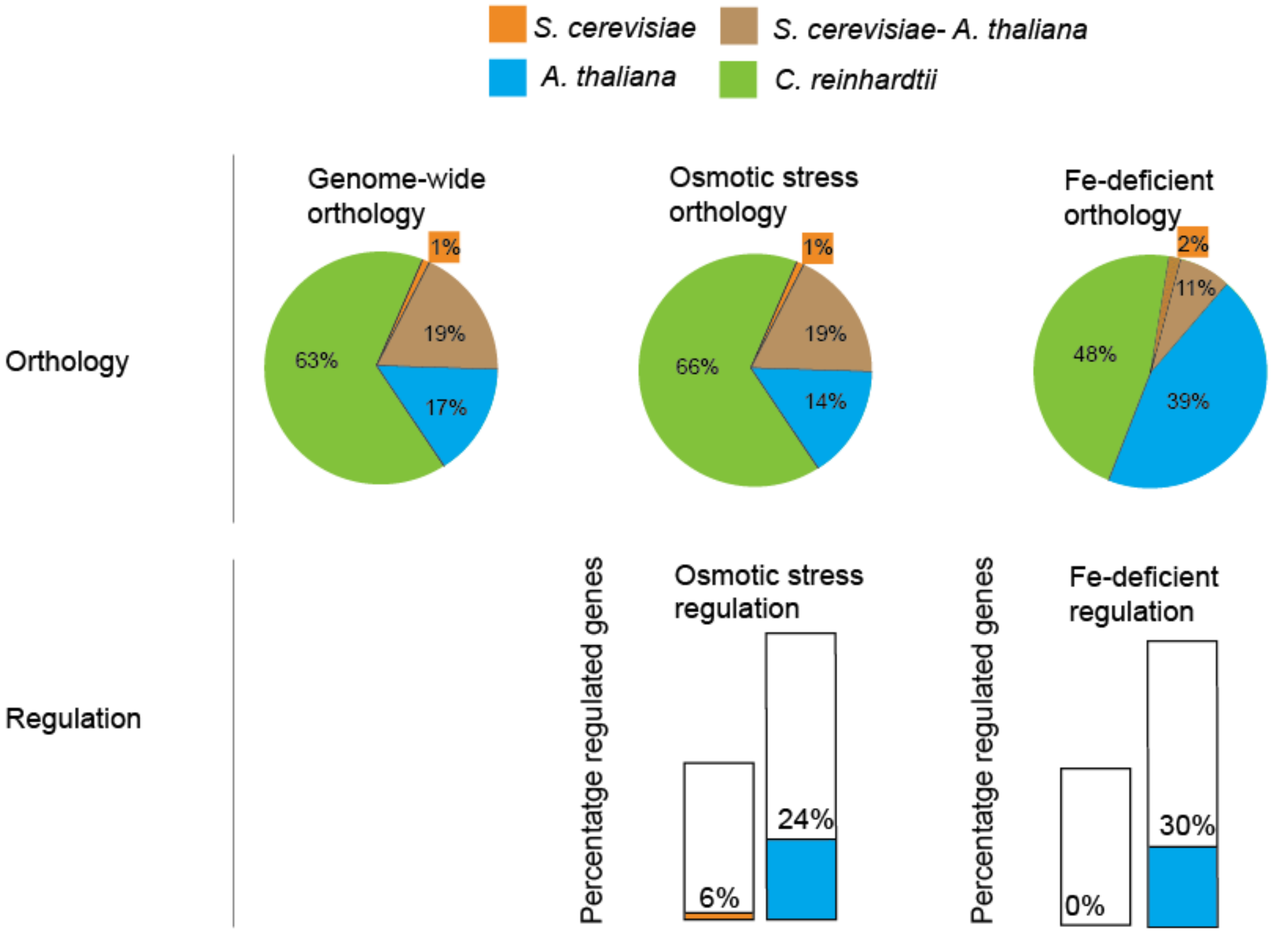

Supplementary Figure 3. Conservation of the osmotic response across kingdoms (related to Figure 1, see also Supplementary Table 2). Pie charts representing the percentatge of orthology for the Chlamydomonas genome (left), osmotically regulated genes (central) or Fe regulated genes (right), with Saccharomyces and Arabidopsis. (left) genome-wide orthology comparison all Chlamydomonas genes with S. Cerevisiae and A. thaliana. (central) Orthology of differentially expressed genes in the Chlamydomonas osmotic time course; (right) Orthology of differentially expressed genes of Chlamydomonas iron-deficiency response; all othologies are based on reciprocal BLAST (e-val > 1E-10) (see Material and Methods and supplementary table S2). Orange, represent genes present in the Chlamydomonas genome with orthologous in S.cerevisiae. Brown, represents genes in the Chlamydomonas genome with orthologs in S. cerevisiae and A. thaliana. Blue represents Chlamydomonas genes with orthologs in A. thaliana. Green represents Chlamydomonas genes without orthologs neither in S. cerevisiae nor A. thaliana. Bars plots represent the percentage of genes differentially expressed upon osmotic stress or iron deficiency in Chlamydomonas with orthologous genes in Saccharomyces (orange bars) or Arabidopsis (blue) responding to the same stress[7,95,96]. (See Material and Methods and supplementary table 2).

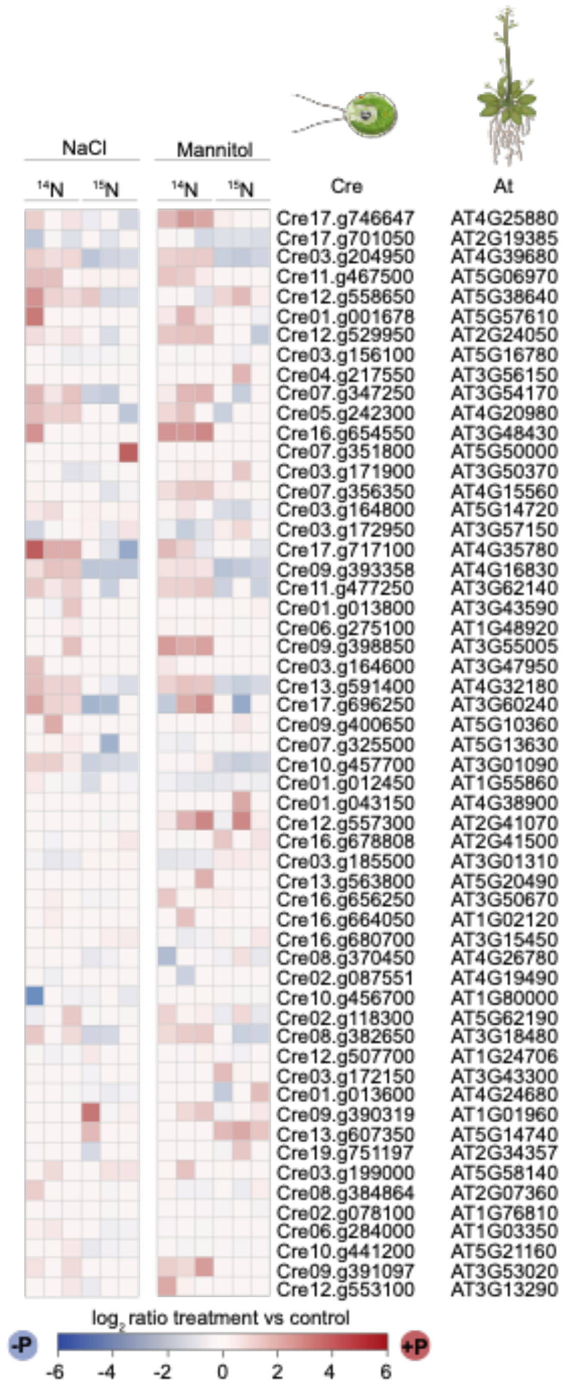

Supplementary Figure 4. Phosphorylated proteins present in Chlamydomonas phosphoproteomics dataset with phosphorylated orthologues in Arabidopsis. (Related Figure 1).

Chlamydomonas peptides showing differential phosphorylation (FC > 1.5, FDR < 0.01) with Arabidopsis orthologous that have previously shown to be differentially phosphorylated upon osmotic related conditions[1,2]. See Supplementary Table 4.

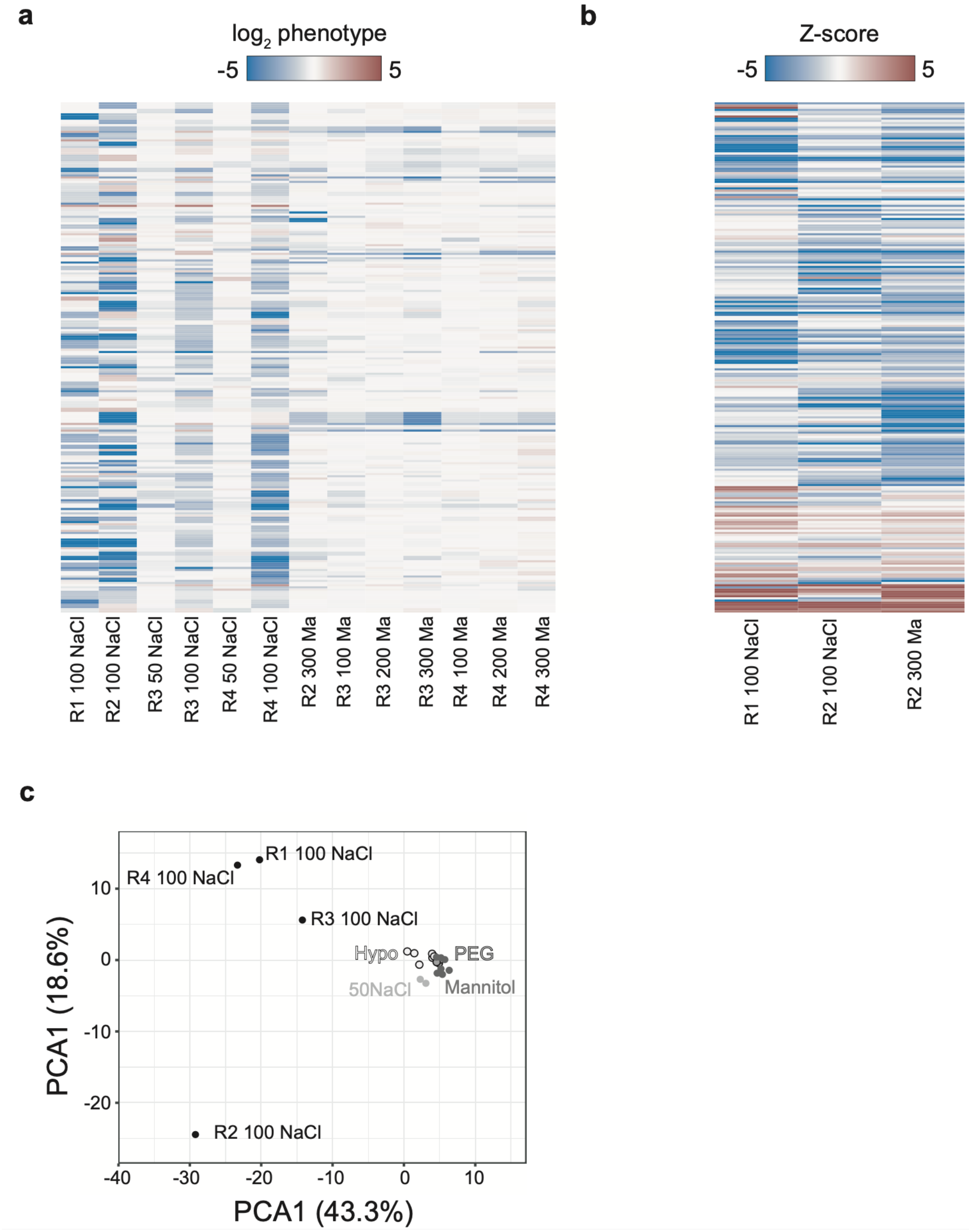

Supplementary Figure 5. Validation of Chlamydomonas mutants with osmotic phenotypes.

A. Median log2 phenotypes resulting from pooled mutant screens based on barcoded reads of hits selected for secondary screens. See Supplementary Table 7.
B. Z-score phenotypes of validated mutants. Z-score results from the quantification of chlorophyll RGB values. See Material and Methods and Supplementary Table 7. Note rows of mutants from A and B match horizontally.
C. PCA of log2 median phenotype from different mutant screen replicas performed.

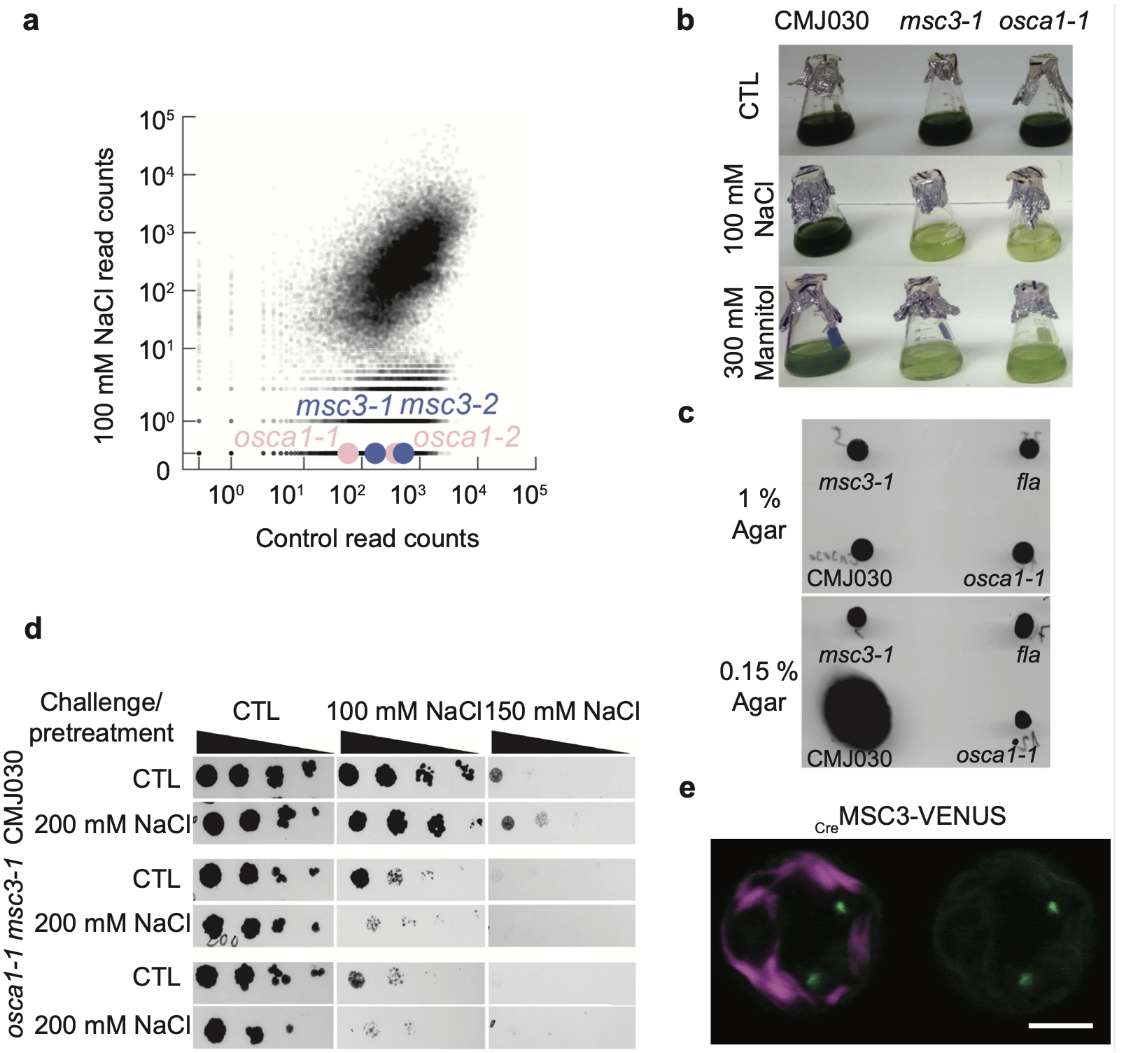

Supplementary Figure 6. Functional conservation of osmoregulatory pathways across the green lineage.

A. Scatter plot comparing barcode reads resulting from pooled screens in control condition and 100 mM NaCl treatment with *osca* and *msl* alleles highlighted. Complete dataset in Supplementary table 5.

B. Growth of wild-type (CMJ030) and *msc3-1* and *osca1-1* under control conditions (CTL), 100 mM NaCl and 300 mM mannitol four days after inoculation.

C. Flagella phenotypes of *msc3-1* and *osca1-1* mutants. (up) strains plated in 1% agar do not show colony differences, (down) strains plated in 0.15% agar show swimming differences based on colony size. As a control we used a flagella less mutant, *fla*. Note that the swimming ability produces larger colonies in 0.15% agar.

D. *Msc3-1* and *osca1-1* mutants show acclimation defects to osmotic stress. Chlamydomonas CTL (CMJ030), *msc3-1* and *osca1-1* cells at the early mid-exponential phase (2E+06 cells/ml) were plated at 10 fold sequential dilutions (control raws, CTL) and Chlamydomonas cells at the exponential phase (2E+06 cells/ml) treated with 200 mM NaCL for 2 hours and plated in 10 fold dilutions (200 mM NaCl).

D. Confocal image of a Chlamydomonas cell expressing MSC3 protein fused to VENUS fluorescent protein. Magenta signal corresponds to chloroplast autofluorescence and green fluorescent signal of VENUS. Scale bar =5 µm.

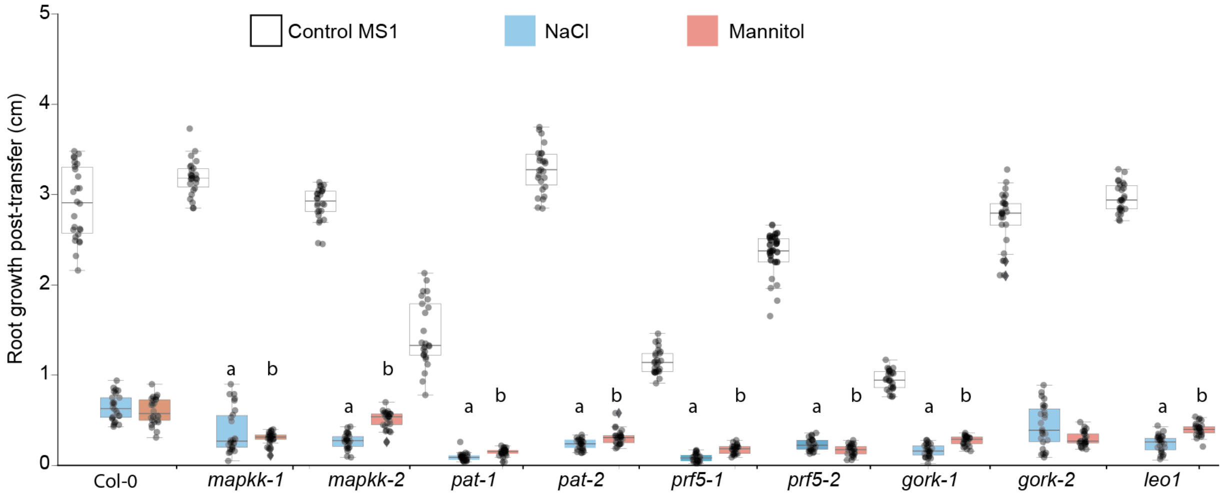

Supplementary Figure 7. Root growth of Arabidopsis mutant roots under different osmotic stress conditions. Root growth of Arabidopsis roots 4 days after transfer to control media (MS1), 140 mM NaCl (NaCl) and 300 mM Mannitol. n=26 for each condition. Center lines show medians and box limits indicate the 25th and 75th percentiles. Whiskers represent minina and maxima. Genotype by treatment interactions significance is shown for each genotype comparing wild-type control and treatment (Col-0) and each mutant control and treatment with two-way ANOVA test. a: represents significant interaction genotype and NaCl treatment ($p<0.05$), b: represents significant interaction genotype and Mannitol treatment. Center lines show medians and box limits indicate the 25th and 75th percentiles. Whiskers represent minina and maxima See Supplementary Table 9 for complete statistical analysis and raw measurements.

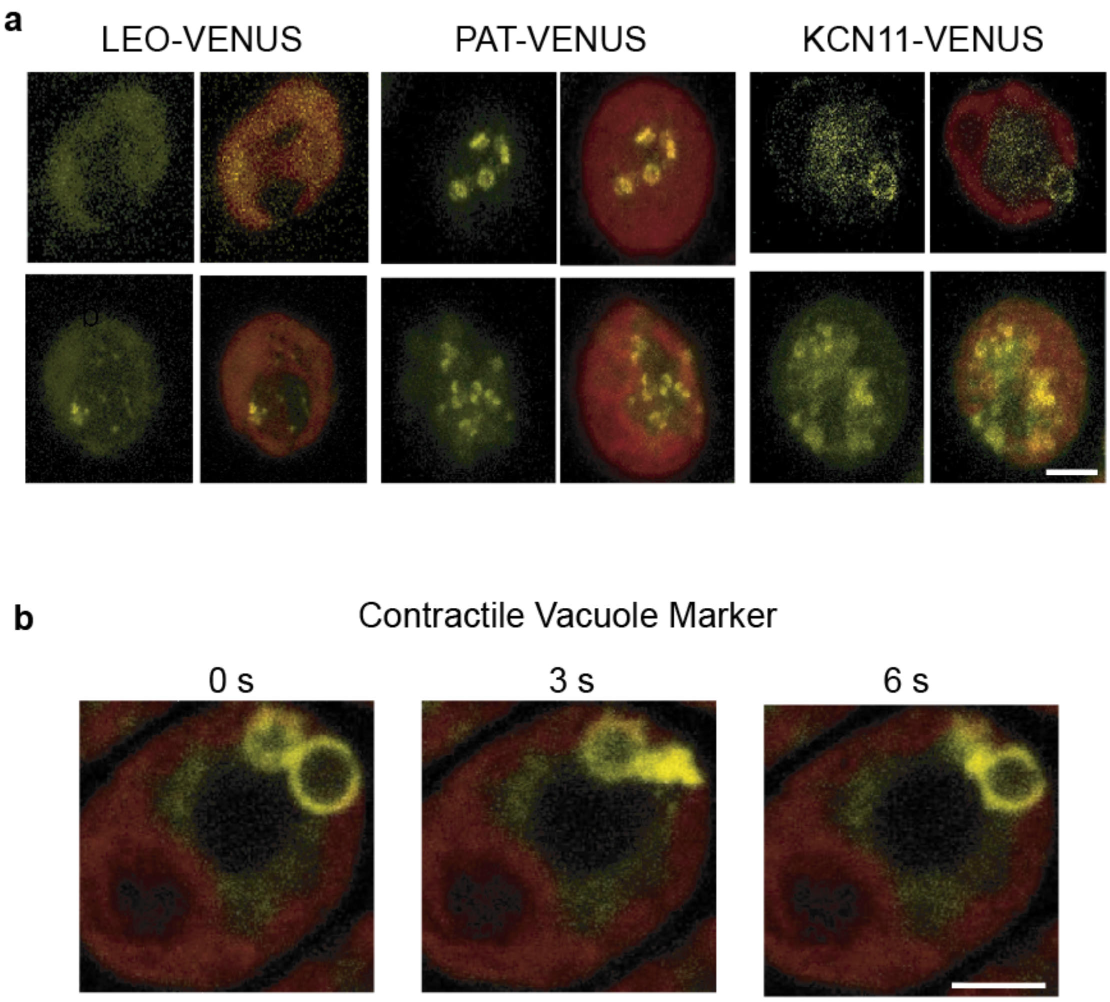

Supplementary Figure 8. Protein localization upon osmotic stress of Chlamydomonas of selected hits identified in the osmotic screens.

A. Confocal images of Chlamydomonas cells expressing proteins fused to VENUS fluorescence protein under control conditions (CTL) and upon treatment with 100 mM NaCl (NaCl). Red colour shows chloroplast autofluorescence and yellow VENUS signal. Scale bar = 5 µm.

B. Confocal images of Chlamydomonas cells expressing a contractile vacuole marker. Red colour shows chloroplast autofluorescence and yellow VENUS signal. Scale bar = 5 µm.

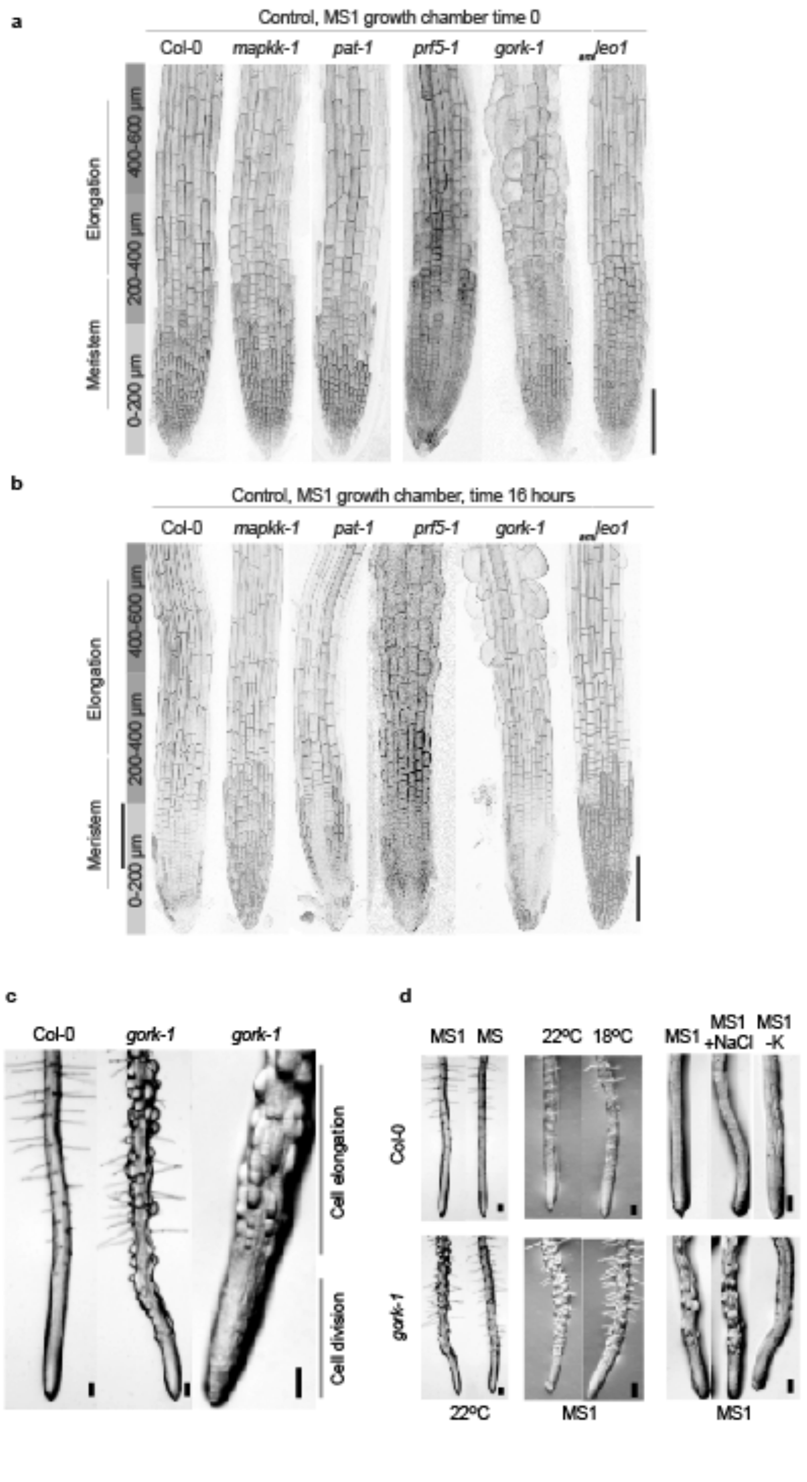

Supplementary Figure 9. Growth dependent accumulation of potassium generates root hair inflated cells.

A. Confocal images of 4 days post germination Arabidopsis primary roots. Time 0 represent the first frame taken during the time-course experiment used to quantify Fig. 2 c-f. Plasma membrane labeled with LTI6b:YFP fluorescence marker. Scale bar = 100 µm.

B. Confocal images of 4 days post germination Arabidopsis primary roots transfer to control media (MS1) for 16 hours in the same growing conditions as Fig. 2 c-f, see methods. Seedlings were grown using the same chambers and growth conditions as roots quantified in Fig. 2 c-f. Plasma membrane labeled with LTI6b:YFP fluorescence marker. Scale bar = 100 µm.

C. Primary roots of 6 days-post germination wild-type (WT) and *gork-1* mutants. Scale bar = 100 µm.

D. Primary root of 6 days-post germination wild-type (WT) and *gork-1* mutants under different sugar concentrations (MS: no sugar; MS1; 1% sucrose), different :temperatures, treatment with 140 mM NaCl (NaCl) and removal of Potassium from the media (-K). Scale bar = 100 μm.

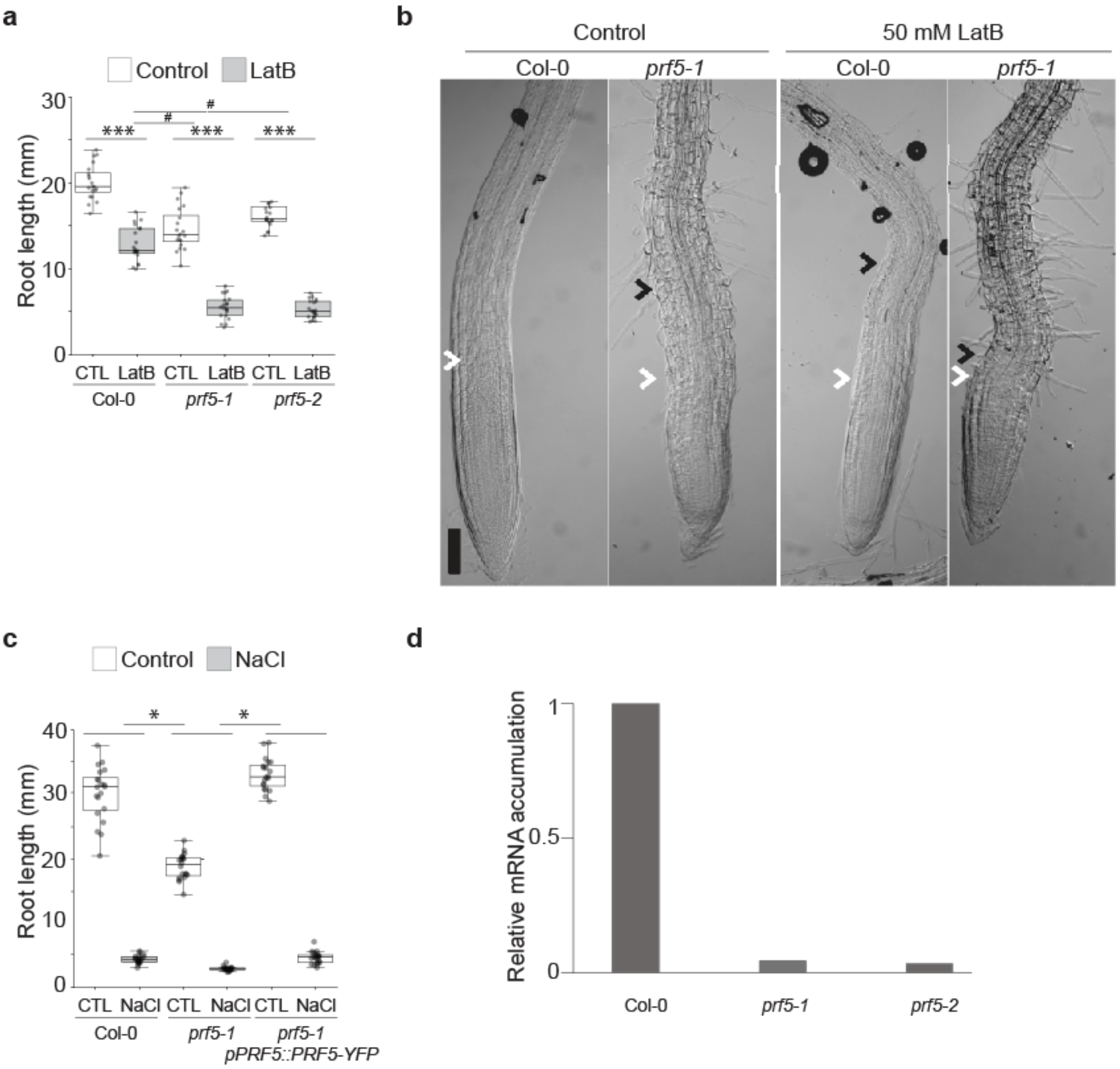

Supplementary Figure 10. Profilin mutants are sensitive to Latrunculin B.

A. Root growth quantification of 6-day old seedlings, wild-type (Col-0) and *prf5*-1 mutant in Control conditions and 50 nM Latrunculin B (LatB). n=20. Center lines show medians and box limits indicate the 25th and 75th percentiles. Whiskers represent minina and maxima. *** indicates significance, one-way ANOVA test of control and treated roots for each genotype p-value<0.001. # indicates significance, two-way ANOVA test of the interaction genotype and treatment p-value<0.001.

B. Root tips of 6 day old seedlings of wild-type (Col-0) and *prf5-1* mutant. The white arrow indicates the boundary between meristematic and elongating cells, black arrow indicates the boundary of the elongation and differentiation root zones. Scale bar = 100 µm.

C. Root growth quantification of 4 days post transfer seedlings to control media or media containing 140 mM NaCl. n=20. Center lines show medians and box limits indicate the 25th and 75th percentiles. Whiskers represent minina and maxima. * indicates significance, two-way ANOVA test between genotype and treatment, p-value<0.001.

D. Relative amount of profilin 5 mRNA accumulation in wild-type (Col-0) and two *prf-5* mutant alleles. Relative mRNA levels between wild-type and profilin mutants as measured by qPCR, UBC30 was used as endogenous control. The graph represents one replica (see Material and Methods and source data for all values).

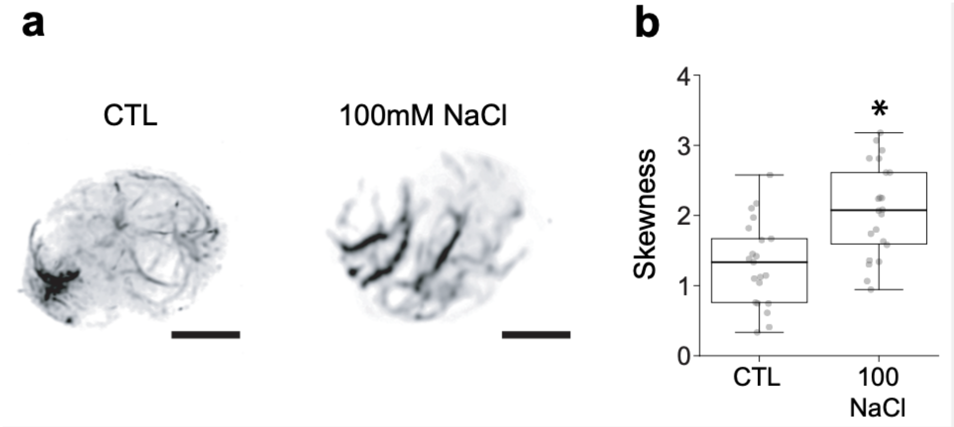

Supplementary Figure 11. Salt stress promotes actin bundling in Chlamydomonas.

A. Actin localization in Chlamydomonas cells under control conditions (CTL) and upon 16 hours treatment with 100 mM NaCl (100 NaCl). Actin filaments were visualized using Lifeact:NEOGREEN. Scale bar = 5 µm.

B. Quantification of actin skewness in Chlamydomonas cells. n=22 cells. Center lines show medians and box limits indicate the 25th and 75th percentiles. Whiskers represent minina and maxima. * indicates significance, one-way ANOVA comparison between untreated and treated cells, p-value=0.000159.

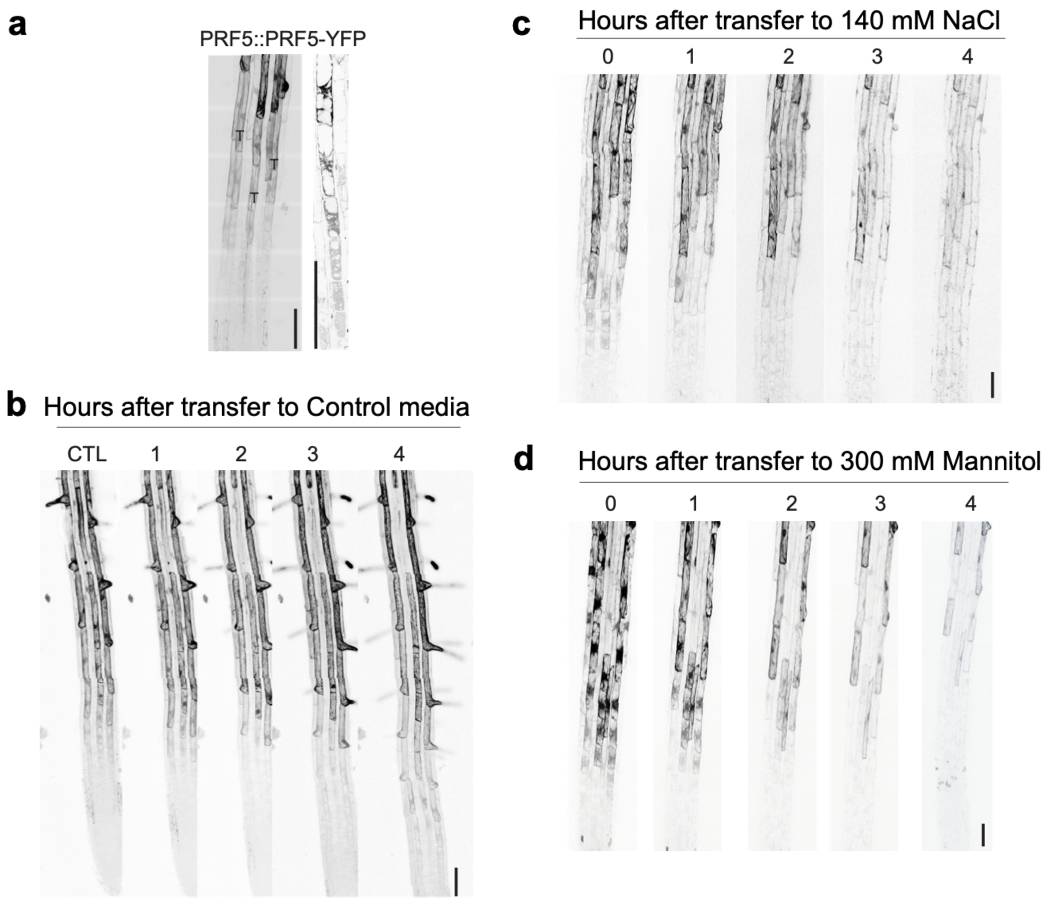

Supplementary Figure 12. Profilin 5 expression is restricted to root hairs and regulated by osmotic stress .

A. Confocal image of Arabidopsis primary root elongation zone expressing *PROFILIN5:PROFILIN5:YFP*. T represents trichoblasts. Left) projection of z-stack, right) single optical section . Scale bar = 100 µm.
B. Confocal images of Arabidopsis primary root elongation zone expressing *ProPROFILIN5:PROFILIN5:YFP* in control media for the indicated hours. Scale bar= 100 µm.
C. Confocal images of Arabidopsis primary root elongation zone expressing *ProPROFILIN5:PROFILIN5:YFP* treated with 140 mM NaCl for the indicated hours. Scale bar= 100 µm.
D. Confocal images of Arabidopsis primary root elongation zone expressing Pro*PROFILIN5:PROFILIN5:YFP* treated with 300 mM mannitol for the indicated hours. Scale bar = 100 µm.

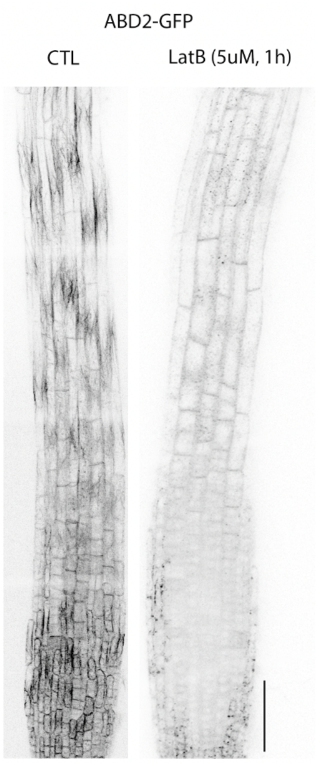

Supplementary Figure 13. Latrunculin B treatment promotes actin depolymerization in root tissues. Confocal images of 5 day-old roots expressing ABD2:GFP, images represent a projection of z-stack sections. Left, roots grown in MS1 control media. Right, roots grown in MS1 control media and transferred to liquid MS media containing 5 µM Latrunculin B (LatB) for 60 min. Scale bars = 100 µm. The images are representive controls used in each experiment were LatB treatment is used.

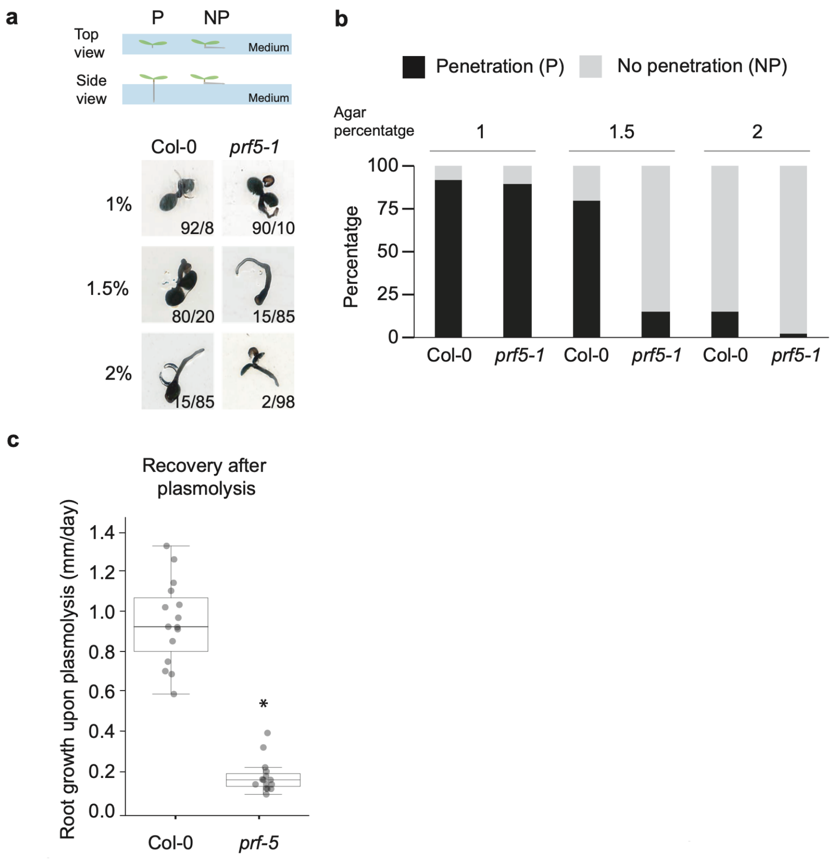

Supplementary Figure 14. Mechanical properties of profilin mutants.

A. Profilin mutant roots have a root penetration phenotype. Wild-type and profilin mutants were planted in plates containing different amounts of agar and root penetration to agar scored. P = penetration, NP = no penetration. The numbers in each image represent number of seedling scored (P/NP).

B. Quantification of penetration of wild-type and *prf5*-1 mutants in different agar concentrations. n=100 seedlings in each condition.

C. Quantification of root growth after plasmolysis of wild-type and *prf5-1* mutants. Plasmolysis was induced by 500 mM Mannitol for 60 minutes and root growth was measured 4 days after recovery in control media. n=15. Center lines show medians and box limits indicate the 25th and 75th percentiles. Whiskers represent minina and maxima. * indicates significance, One way ANOVA comparison between wild-type (Col-0) and *prf-5-1* mutant, p-value = 1.7039 $x10^{-13}$.

References

1. Bhaskara, G. B., Nguyen, T. T., Yang, T.-H. & Verslues, P. E. Comparative Analysis of Phosphoproteome Remodeling After Short Term Water Stress and ABA Treatments versus Longer Term Water Stress Acclimation. *Front. Plant Sci.* **8**, 523 (2017).
2. Wang, P. *et al.* Quantitative phosphoproteomics identifies SnRK2 protein kinase substrates and reveals the effectors of abscisic acid action. *Proc. Natl. Acad. Sci. U. S. A.* 110, 11205–11210 (2013).
