# Supplementary material for: Multi-omics analysis of green lineage osmotic stress pathways unveils crucial roles of different cellular compartments
Source: Nat Commun. 2024 Jul 16;15:5988. doi: 10.1038/s41467-024-49844-3 (PMC11252407; doi:10.1038/s41467-024-49844-3)
Supplement: Supplementary file 3 — Description of Additional Supplementary Files [file 41467_2024_49844_MOESM3_ESM.pdf]

Description of Additional Supplementary Files

Supplementary Data 1. Chlamydomonas transcriptomic response to osmotic stress. Raw data from the Chlamydomonas response to osmotic stress, NaCl and mannitol. Data can be additionally found through the National Center for Biotechnology Information Sequence Read Archive (SRA), GSE260814 [https://www.ncbi.xyz/geo/query/acc.cgi?acc=GSE260814].

Supplementary Data 2. Analysis of the Chlamydomonas transcriptomic response to osmotic stress.a) List of differentially express genes log2 fold change greater than 2 and FDR<0.01. b) Gene onthology analysis of differentially expressed genes in each timepoint. Analysis performed with BINGO plug-in in Cytoscape. Source Figure 1 a and Supplementary data 1 f. c) Overlap ABA regulated genes in Arabidopsis and Chlamydomanas osmotic timecourse (Supplementary Figure 1 e). d) Enrichment of Flagella genes in Chlamydomonas osmotic timecourse. e) Conservation NaCl and Fe response in Chlamydomonas-Arabidopsis-Yeast, related Supplementary Figure 3. f) Conservation Chlamydomonas-Arabidopsis-Yeast transcriptomic response to osmotic stress (Related to supplementary figure 3).

Supplementary Data 3. Chlamydomonas phosphoproteomic response to omsotic stress. Raw Data from the Chlamydomonas response to osmotic stress, NaCl and Mannitol. This data is also available throught the Center for Computational Mass Spectometry, Mass Spectrometry Interactive Virtual Environment (MassIVE) (https://massive.ucsd.edu/ProteoSAFe/static/massive.jsp), dataset ID; MSV000094492.
[https://massive.ucsd.edu/ProteoSAFe/dataset.jsp?task=3e301feb87904983b4ccd5d9e949350b].

Supplementary Data 4. Analysis of the Chlamydomonas phosphoproteomic response to osmotic stress. a) Annotation peptides differentially phosphorylated (Fold change>2, FDR<0.01) upon 100 mM NaCl and 300 mM Mannitol. Related Figure 2. b) Chlamydomonas differentially phopshorylated peptides with orthologous proteins in Arabidopsis differentialy phosphorylated. Data used in Supplementary Figure 4. c) Chlamydomonas differentially phopshorylated peptides (FC>1.5 FDR<0.01). d) Raw data used for Figure 2. e) Phosphosites of differentially phosphorylated proteins displayed in Figure 2.

Supplementary Data 5. Chlamydomonas mutants sensitive to osmotic stress. a) Osmotic screens normalized log2 phenotypes for each mutant screen. b) Gene Ontology analysis of osmotic screens.

Supplementary Data 6. Chlamydomonas high confidence osmotic hits. a) Adjusted p-values of high confidence Chlamydomonas hits resulting from Barcoded mutant screens. Including Raw data for Figure 3 b. b) Chlamydomonas mutants high confidence hits phenotype, median phenotype. c) Annotation of Chlamydomonas high confidence hits from osmotic screens. d) Phenotype of Chlamydomonas osmotic screens high confidence genes FDR<0.3 in 121 conditions described in (Vilarrasa-Blasi, Fauser et al 2022).

Supplementary Data 7. Chlamydomonas secondary screens. a) log2 median phenotype of mutants validated in secondary screens. b) Z-scores of validated genes in secondary mutant screens.

Supplementary Data 8. Protein-Protein interactions of Osmotic hits. a) Protein-protein interactions of Arabidopsis orthologous genes to high confidence Chlamydomonas hits from all osmotic screens. b) High confidence Arabidopsis orthologous hits with two or more shared interactors. c) Annotation of the 32 high confidence Arabidopsis orthologous hits with two or more interactors.

Supplementary Data 9. Root growth of Arabidopsis mutants. Root length 4 days posttransfer to 140 mM NaCl (NaCl) or 300 mM Mannitol (Ma) of different mutant alleles and wild-type plants.

Supplementary Data 10. Primers used in thi study. List of primers used in this manuscript**.**

Supplementary Data 11 Strains used in this study. Chlamydomonas and Arabidopsis genotypes used in this manuscript.
